# An Uncommon Case of Bilateral Peroneal Nerve Palsy following Delivery: A Case Report and Review of the Literature

**DOI:** 10.1155/2014/746480

**Published:** 2014-08-11

**Authors:** Kristen Bunch, Erica Hope

**Affiliations:** ^1^Department of Gynecologic Oncology, Walter Reed National Military Medical Center, Bethesda, MD 20889, USA; ^2^Department of Obstetrics and Gynecology, Walter Reed National Military Medical Center, Bethesda, MD 20889, USA

## Abstract

Peroneal nerve palsy is an infrequent but potential complication of childbirth. Bilateral peroneal palsy is particularly rare following delivery with few reported cases. A 38-year-old gravida 1, para 0 underwent a prolonged second stage of labor, was diagnosed with an arrest of descent, and subsequently underwent an uncomplicated primary cesarean section. The patient was diagnosed with bilateral peroneal neuropathy four days after delivery. By two months postpartum, her foot drop had improved by 85% and the remainder of her symptoms resolved. Awareness of the risks of a peroneal neuropathy as well as implementation of preventive measures is important for members of the delivery team. Regional anesthesia during labor is a risk factor for the development of a peroneal neuropathy.

## 1. Introduction

The most common lower extremity mononeuropathy is a peroneal nerve palsy [1]. In obstetrics, sciatic and femoral neuropathies are much more common and typically associated with an instrument-assisted delivery; however, peroneal nerve palsy is another less commonly encountered peripheral neuropathy [2–4]. Causes of peroneal nerve injury during childbirth include prolonged external knee compression from stirrups or hand positioning, knee hyperflexion, and prolonged squatting. Several cases of unilateral peroneal neuropathy following childbirth have been reported [4–7]; however, bilateral peroneal nerve palsy is rare and few cases following delivery have been reported [8].

**Figure 1:**
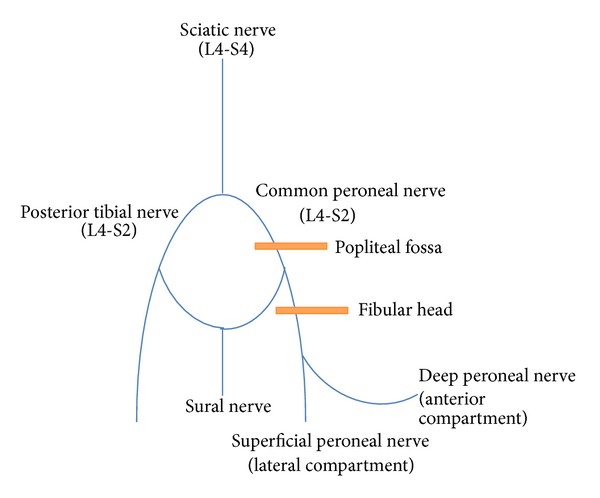
Anatomy of the peroneal nerve. The sciatic nerve branches into the tibial and common peroneal nerves superior to the popliteal fossa. The orange bands represent areas of potential injury to the peroneal nerve. The peroneal nerve is most commonly injured during childbirth from prolonged or forceful external compression to the fibular head while grasping and flexing the knee when pushing in the semi-Fowler-lithotomy position. The superficial location of the peroneal nerve as it courses around the fibular head increases the risk of injury or trauma to the peroneal nerve.

A review of the anatomy traversed by the peroneal nerve lends greater appreciation to its vulnerability to injury. The common peroneal nerve branches from the sciatic nerve at the level of the popliteal fossa, continues along the medial aspect of the biceps femoris muscle, and passes superficially over the lateral head of the gastrocnemius muscle. It then courses laterally around the fibular head adjacent to the periosteum. The superficial location of the nerve at the fibular neck predisposes it to injury from compression or trauma at this site, particularly given the typical hand positioning while pushing during labor. The common peroneal nerve then travels between the fibula and the peroneal longus muscle, dividing into the superficial and deep branches (Figure 1) [8]. The superficial branch is primarily sensory, innervating the dorsum of the foot and anterolateral aspect of the lower leg, and also supplies motor innervation to the peroneus longus and brevis muscles. The deep peroneal nerve primarily innervates the muscles of the foot and ankle responsible for eversion and dorsiflexion and provides sensory innervation to the first web space [1, 9].

## 2. Case Presentation 

A previously healthy 38-year-old gravida 1, para 0 was admitted to labor and delivery in active labor at 38 + 5 weeks estimated gestational age. Her prenatal course was complicated by an episode of nephrolithiasis at 28 weeks gestation and gestational hypertension that was diagnosed on her admission. The patient received epidural anesthesia for pain control. She progressed to complete dilation and effacement and began pushing efforts at +1 station. During the second stage of labor, different positioning techniques were utilized while pushing. No further descent of the fetal head was made over the course of four hours despite adequate contractions and active pushing, and she underwent an uncomplicated primary low transverse cesarean section for arrest of descent. She was discharged to home on postoperative day two with no issues noted before discharge.

The patient was seen in clinic on postoperative day four for a blood pressure check and complained of difficulty ambulating since delivery, requiring assistance for walking, and mild bilateral lower extremity edema. Upon reviewing her delivery and hospital course, the patient did report that she had some weakness when walking prior to hospital discharge but her symptoms had progressed to the point of needing ambulatory assistance. On examination, she was noted to have a steppage gait, decreased strength of the left lower extremity, and intact sensation to the bilateral lower extremities with bilateral foot drop. The patient also complained of right-sided lower back and buttock pain with movement and intermittent “lightning bolt” sensation to thighs.

She was readmitted on postoperative day four for hypertensive control as well as additional evaluation of her lower extremity neuropathy. Anesthesiology and neurology were also consulted given her neuropathy symptoms. The neurology exam was notable for a wide based gait secondary to weakness, grossly intact sensation, and mild weakness on ankle dorsiflexion. Magnetic resonance imaging of the lumbar spine was obtained to evaluate for epidural hematoma, which showed no remarkable findings. Her bilateral plexopathy was felt to be secondary to a common peroneal nerve injury, likely from prolonged pressure to the lateral knee resulting in compression of the peroneal nerve between the fibular head, the biceps tendon, and gastrocnemius muscle during her prolonged pushing phase. Physical therapy was consulted and further evaluation with electromyographic studies was also recommended. She was discharged to home on postoperative day six with adequate blood pressure control on oral antihypertensive therapy and subjectively and objectively improved strength in her bilateral lower extremities.

The patient was evaluated on postoperative day 7 reporting significant improvement in pain and ambulation since hospital discharge and declined physical therapy at that time. On postoperative day 14, the patient was again seen in clinic and reported no change in symptoms or gait and a physical therapy consult was ordered as well as a follow-up appointment with neurology for an electromyographic study. The patient did not follow up with physical therapy or neurology and was subsequently seen on postoperative day 21 when she again reported significant improvement in her gait and near resolution of her foot drop. Our patient recovered spontaneously without any therapeutic intervention and was noted to have a normal neurological exam, 5/5 strength on dorsiflexion bilaterally, and approximately 85% resolution of her foot drop by 7 weeks postpartum.

## 3. Discussion

Neurologic injury during childbirth has long been recognized as a potential complication, with the lateral femoral cutaneous nerve as the most common obstetric-related nerve injury (Table 1) [9]. Peroneal nerve injury is less commonly encountered and typically unilateral; the incidence of bilateral peroneal neuropathy following childbirth is extremely rare. According to a large review by Wong et al. evaluating the incidence of lower extremity neuropathies after childbirth, the incidence was found to be 0.92%. Factors associated with lower extremity nerve injury in this study were nulliparous women and a prolonged second stage of labor [2]. Most cases of bilateral peroneal neuropathy associated with childbirth occur in developed countries from prolonged mechanical external knee compression and forceful knee flexion. Additionally, nerve injury can result from an extended period of low pressure as well as a short interval of high pressure [4]. Although the vast majority of neurologic injuries associated with childbirth are intrinsic obstetric palsies, neuraxial anesthesia is a risk factor for an obstetric-related neurologic injury [9]. Regional anesthesia, in particular, increases the risk for developing a peroneal neuropathy as it blocks sensation to the lower extremities and, therefore, recognition of an impending nerve injury such as pain or altered sensation.

Following placement of neuraxial anesthesia and during delivery, it is imperative that maternal positioning be considered and reassessed throughout the labor course. A study by Bsteh et al. evaluating lower extremity neuropathy associated with lithotomy positions in a surgical patient population identified that the frequency of neuropathy increased after 2 hours of lithotomy positioning; however, the duration and amount of pressure necessary to cause injury are not known [10]. Women with neuraxial anesthesia change positions less often during labor and more frequently push in the lithotomy position predisposing them to lower extremity neuropathy [9].

Individuals with a peroneal nerve injury often present with complete or partial foot drop in addition to a change in gait, such as a steppage gait. Reflexes are spared, as are plantar flexion and inversion of the ankle. However, weakness of the ankle dorsiflexors and evertors is common and results in the characteristic foot drop [1]. Altered sensation to the dorsum of the foot but not the plantar aspect supports the diagnosis of a peroneal neuropathy; thus, a thorough sensory exam will assist in localizing the lesion.

Prolonged or forceful hand positioning by the patient or other birth attendants has been described as the cause of peroneal neuropathy following childbirth [3, 4]. Although the peroneal nerve is typically injured from excessive pressure at the fibular head, the nerve is also susceptible to injury from excessive pressure at the distal femur. Precaution measures were taken with this patient to prevent a nerve palsy injury, such as frequent position changes and relieving pressure on the knees between pushing. However, the bilateral peroneal neuropathy demonstrated in this patient was most likely injured from prolonged, excessive pressure on the peroneal nerve near the fibular neck during knee abduction or as a result of stretch or traction on the nerve at the level of the knee. Additionally, regional anesthesia prohibited the patient from recognizing any symptoms of an impending nerve injury.

Electrodiagnostic studies are helpful in localizing and confirming the site of nerve injury. The extent of nerve injury can also be defined by electrodiagnostic studies as well as determining recovery prognosis. Although the optimal timing for such studies is unknown, testing between 2 and 12 weeks following the injury has been suggested as a meaningful time to assess extent of nerve damage. Electrodiagnostic studies also aid in classifying nerve injuries as axonal or demyelinating. Demyelinating injuries are associated with a conduction block and no signs of denervation, while axonal lesions do not demonstrate a conduction block. Additionally, demyelinating nerve injuries have a better prognosis than axonal lesions [10].

Initial treatment of a peroneal neuropathy is typically conservative management and includes a variety of interventions such as stretching, range of motion exercises, and strength training. Neuropathic pain is a common complaint and may be treated with oral analgesics, capsaicin, topical lidocaine, opioids, or heat and ice treatments. Caution must be taken to ensure the skin is protected when using heat and ice therapy. Depending on the severity of the lesion and extent of symptoms, ankle foot orthosis may be necessary for toe clearance during ambulation [8].

Peroneal neuropathy is an infrequent complication from childbirth; however, awareness of this particular neuropathy is important for labor and delivery teams, since it is a preventable injury. Furthermore, since the majority of women in developed countries use regional anesthesia for pain control during labor and may not appreciate symptoms of an impending nerve injury due to sensory blockade, there is a large population at risk for injury. Most importantly, a peroneal nerve injury can be prevented in a laboring patient by employing frequent maternal position changes as well as releasing external pressure on the knees while not actively pushing. Prompt recognition of a peroneal neuropathy is important in ensuring appropriate treatment, preservation of maximum function, and resolution of the injury as well as prevention of any further injury. Labor and delivery teams must remain cognizant of this preventable childbirth injury, the associated risk factors, and preventive measure to avoid maternal morbidity.

## Figures and Tables

**Table 1:** Obstetric palsies.

Nerve	Nerve roots	Sensory deficit	Motor deficit
Lumbosacral plexus	L1-S4	Lateral leg Dorsum of foot	Foot dorsiflexion and eversion (foot drop)
Obturator nerve	L2-L4 (anterior division)	Medial thigh Knee	Thigh adduction
Femoral/saphenous nerve	L2-L4 (posterior division)	Anterior thigh Medial leg Foot	Hip flexion Knee extension Patellar reflex
Lateral femoral cutaneous nerve	L2-L3	Anterolateral thigh “meralgia paresthetica”	None
Sciatic nerve	L4-S4	Buttocks Posterior thigh/leg/foot	Knee flexion
Posterior tibial nerve	L4-S2	Sole of foot	Foot plantar flexion and inversion
Common peroneal nerve	L4-S2	Anterolateral leg Dorsum of foot	Foot dorsiflexion and eversion (foot drop)

Table adapted from Wong, 2010 [9].
